# A systematic review exploring pre-COVID-19 telehealthcare models used in the management of patients with rheumatological disease

**DOI:** 10.1093/rap/rkab073

**Published:** 2021-11-13

**Authors:** Alexandra Jayne Nelson, Marina Ellen Anderson

**Affiliations:** 1 Institute of Ageing and Chronic Disease, University of Liverpool, Liverpool; 2 Lancaster Medical School, Lancaster University, Lancaster, UK

**Keywords:** Telehealthcare, telemedicine, telerheumatology, rheumatic disease, systematic review

## Abstract

**Objective:**

The aim was to assess the use of telehealthcare in rheumatology before coronavirus disease 2019 (COVID-19), to which future comparisons of newer interventions adapted during the crisis can be made.

**Methods:**

We performed a registered systematic literature search using MEDLINE, EMBASE, CENTRAL and PubMed databases. All full-length articles comparing telehealthcare delivery models with standard care (face-to-face consultation) in the management of patients with rheumatic conditions were assessed for inclusion.

**Results:**

A total of 4809 studies were identified; 108 studies were suitable for review by full text, and 13 studies were appropriate to be included in this review. Five studies (38%) included patients with RA, four studies (31%) included patients with mixed disease cohorts, two studies (15%) included patients with OA, one study (8%) included patients with JIA, and one study (8%) included patients with FM. Six studies (46%) used telephone consultation, three studies (23%) used mixed method communication, three studies (23%) used videoconferencing, and one study (8%) used website-delivered telecommunication as their method of telehealthcare delivery. Overall, seven studies (54%) identified the telehealthcare intervention to be an effective method of consultation, and six studies (46%) identified the telehealthcare intervention as non-inferior when compared with standard care.

**Conclusion:**

Current evidence for telehealthcare in rheumatology is lacking, and the evidence for effectiveness is limited by methodological bias and clinical heterogeneity of telehealthcare interventions, preventing definitive inferences. Scrutinous assessment of the current telehealthcare interventions used during COVID-19 is required to accommodate recommendations and guideline reviews directed from international working groups.

Key messages

- There is a lack of robust evidence evaluating the use of telehealthcare in rheumatology prior to COVID-19.
- Telehealthcare has become an essential service in the delivery of health care during the COVID-19 pandemic.
- Future research objectively assessing the effectiveness of post-COVID-19 practices of telehealthcare interventions are required.

## Introduction

Recent advancements in the delivery and utilization of information and communication technologies (ICTs) and the growing prevalence of technological resources worldwide have led to an increased application of telehealthcare services [1–3]. ICTs used in the delivery of telehealthcare have the potential to improve health-care accessibility, cost effectiveness and quality of health care [3]. The diagnostic and disease management capabilities of telehealthcare have increased greatly following the increased sophistication of ICTs of the 21^st^ century. There are specific factors that have supported the development of telehealthcare over the years including the growing prevalence of chronic diseases, financial shortages for health-care resources and a greater demand for flexibility of care [3]. In the past, telehealthcare has largely been used for remote and rural communities, increasing accessibility of health care where it was otherwise limited. However, there is increasing demand for wider application of telehealthcare throughout all domains of health care, including the management of patients with rheumatic conditions, increasing the number of remote follow-up consultations and home monitoring in this setting [4].

The global coronavirus disease 2019 (COVID-19) crisis rapidly accelerated pressure on health-care systems worldwide [5]. Telehealthcare has become an essential and pragmatic service for patients, helping to mitigate the spread of COVID-19, preserve hospital resources and provide patient care, while maintaining the government-imposed ‘social distancing’ restrictions [6]. COVID-19 has introduced a need for policy guidelines for effective transfer of care, imposed by the forced reduction of face-to-face consultation and increase in remote working. Reducing the risk of exposure of patients and health-care workers to COVID-19 is the aim of telehealthcare practices during this time. This is particularly important in patients with rheumatological disease. Patients with rheumatological disease, particularly those on immunosuppressant therapy, are potentially at increased risk of COVID-19 morbidity and developing the severe consequences of the disease. Subsequently, these patients were advised to shield during the pandemic [5]. Additionally, during a time when health-care finances are being stretched to the limit, telehealthcare practices can reduce costs and have financial advantages compared with face-to-face consultations [6]. During the COVID-19 crisis, distance has been eliminated as a determining factor for the application of telehealthcare practices, introducing a wider scope of application for telehealthcare.

Even before COVID-19, rheumatology health-care systems were struggling to meet the demand for face-to-face clinics [6]. There are, however, limitations to the use of telehealthcare, particularly within rheumatology, relating to effective patient examination and disease monitoring, which might become difficult via remote communication techniques [7]. The time has now come, ever more than before, to re-evaluate the purpose of outpatient care in rheumatology and align those objectives with modern-day living and expectations. The aim of this review is to assess the use of telehealthcare in the management of rheumatic diseases before COVID-19, to which future comparisons of newer interventions adapted during the crisis can be made.

## Methods

### Protocol and registration

This systematic review was conducted in accordance with The Cochrane Collaboration principles of Systematic Reviews and reported following the Preferred Reporting Items for Systematic Reviews and Meta-Analyses (PRISMA) guidelines [8, 9]. The protocol for this systematic review outlining the methods for study inclusion and data analysis were pre-specified and registered with the International Prospective Register of Systematic Reviews (PROSPERO) database (registration number: CRD42020180695).

### Eligibility criteria

#### Study characteristics

All full-length articles comparing telehealthcare delivery models with standard care (face-to-face consultation) in the management of patients with rheumatic conditions, before changes made for COVID-19, were assessed for inclusion. Non-randomized, randomized, prospective and retrospective studies published in the English language were considered for review. Case studies, editorials, letters, practice guidelines, grey literature, abstract-only reports, reviews and commentaries were excluded from this review because it can be difficult to appraise study quality from these sources, and unpublished studies are likely to be harder to source. Abstract-only reports were initially considered but excluded on the basis that differences often occur between data reported in conference abstracts and their corresponding full reports, limiting the accuracy of results obtained from these sources. Studies with <6 week duration were excluded because this study period might not allow for repeated follow-up, and a time bias may exist within shorter study periods that would not detect the true result of the telehealthcare intervention. Studies including patients admitted to hospital during the study or studies with patients receiving both standard care and telehealthcare delivery were excluded, unless standard care was provided at baseline and the end of the study.

#### Patient characteristics

All patients of any age who had been diagnosed with a rheumatic condition and were managed by rheumatology professionals were included. Studies using trained rheumatologists, advanced nurse practitioners, physician associates or any other allied health-care professional involved in the management of rheumatic conditions as telehealthcare facilitators and standard care providers were included. Rheumatic conditions to be excluded from this review are infective rheumatic diseases (gonococcal arthritis, tuberculosis, osteomyelitis etc.), diseases associated with rheumatic conditions (ulcerative colitis, uveitis etc.), traumatic or neurodegenerative disorders and patients with arthritis relating to drug reactions.

#### Intervention and comparators

Telehealthcare interventions included all forms of remote consultation, including synchronous and asynchronous forms, used in the management of patients with rheumatic disease. All telehealthcare interventions must have been implemented before the changes to health-care delivery as a result of COVID-19. The comparator was standard care throughout, including face-to-face consultation, management and follow-up of patients with rheumatic disease. Studies must include both of these cohorts to be included. Studies of telephone triage systems, website or mobile applications used in patient self-management were included if the study outcomes evaluated the effect of telehealthcare on disease management.

#### Outcome measures and synthesis

The primary outcomes of this review were to determine the types of telehealthcare models that have been used in the management of rheumatological disease and the disease states that have been studied. A study conclusion was extrapolated from each study regarding telehealthcare effectiveness based on the authors’ conclusion: this was identified as being effective, non-inferior or inconclusive in achieving the outlined outcome measures in individual studies compared with standard care. Secondary outcomes included: the proportion of telehealthcare studies that were effective and the measured outcomes of telehealthcare effectiveness, the purpose of telehealthcare intervention, telehealthcare facilitator grade, association of telehealthcare with patient satisfaction and cost effectiveness, and patient attrition rates in telehealthcare studies. Outcomes were summarized in tabular format and collated, providing a narrative synthesis of the existing literature describing details of the types of telehealthcare interventions studied and the authors’ conclusions from these studies.

### Information sources and search strategy

A comprehensive broad literature search was conducted with the assistance of a health information specialist. Databases were accessed via National Institute of Clinical Excellence (NICE) Healthcare Database Advanced Search (HDAS) using OpenAthens in early April 2020. Studies were identified by searching Medical Literature Analysis and Retrieval System Online (MEDLINE), Excerpta Medica database (EMBASE), the Cochrane Central Register of Controlled Trials (CENTRAL) and PubMed. A combination of key words and Medical Subject Headings (MeSH) terms (MEDLINE, EMBASE and CENTRAL) relating to telehealthcare and rheumatology were used. Key words used in the search strategy included ‘exp TELEMEDICINE/’, ‘telemedicine’, ‘telehealth’, ‘telecommunication’, ‘telehealthcare’, ‘tele consult’, ‘tele* consult*’, ‘phone* consult*’, ‘telemed*’, ‘remote consult*’, ‘remote communicat*’, ‘remote access’, ‘remote management’, ‘telecare’, ‘videoteleconfer*’, ‘VTC’, ‘Ehealth’, ‘Econsult’, ‘Interactive’, ‘Video confer*’, ‘Twoway’, ‘Asynchronous consult’, ‘Synchronous consult’, for telehealthcare techniques. A full description of the search strategy including the rheumatological conditions used in HDAS is provided (Supplementary Table S1, available at *Rheumatology Advances in Practice* online). No *a priori* study publication date range restrictions were used.

### Data extraction and study selection

Database searches were carried out by one reviewer. The results of studies identified from the search strategy were exported to Endnote X9 (Clarivate Analytics, Philadelphia, PA, USA) and any duplicate entries were removed. All citations were then imported into the Covidence systematic review platform (Veritas Health Innovation, Melbourne, Victoria, Australia). Two reviewers independently screened the titles and abstracts of the identified studies from the search strategy, and any potentially relevant studies were then screened against the eligibility criteria. Any disagreements or conflicts in this screening process were resolved by discussion between the two reviewers, and unresolved conflicts were arbitrated by a third reviewer if necessary. The corresponding authors of studies were contacted if clarification regarding the methodology and/or data of a study was required. Reference lists of all included studies, review articles and sources known to the authors were screened manually to identify additional studies. Data were extracted independently by one researcher and documented in tabular format for analysis, then reviewed by a second researcher. Information including the following characteristics was extracted from studies: (1) study design/methodology, including title, authors, journal, publication date, study type, study period and number of participants; (2) population characteristics (age, sex and diagnosis); (3) health-care setting (location, telemedicine mode, facilitator grade, communication methods and duration of follow-up); (4) recruitment procedures; and (5) the outcome measures and reported key findings in the study.

### Assessment of bias

Quality of each study was evaluated by applying the Revised Cochrane Risk of Bias Tool for randomized controlled trials (RoB 2) [10]. Bias was assessed as a judgement (high, low or some concerns) into the following domains: randomization process, effect of assignment to intervention, effect of adhering to the intervention, missing outcome data, measurement of outcome, selection of reported result and overall bias. Non-randomized trials were assessed using a modified Newcastle–Ottawa Scale (NOS) for cross-sectional studies and/or cohort studies. NOS is judged on three broad domains: the selection of study groups, comparability and ascertainment of outcome of interest, scored using a star (*) point system (maximum 10*) [11].

## Results

**Fig. 1:**
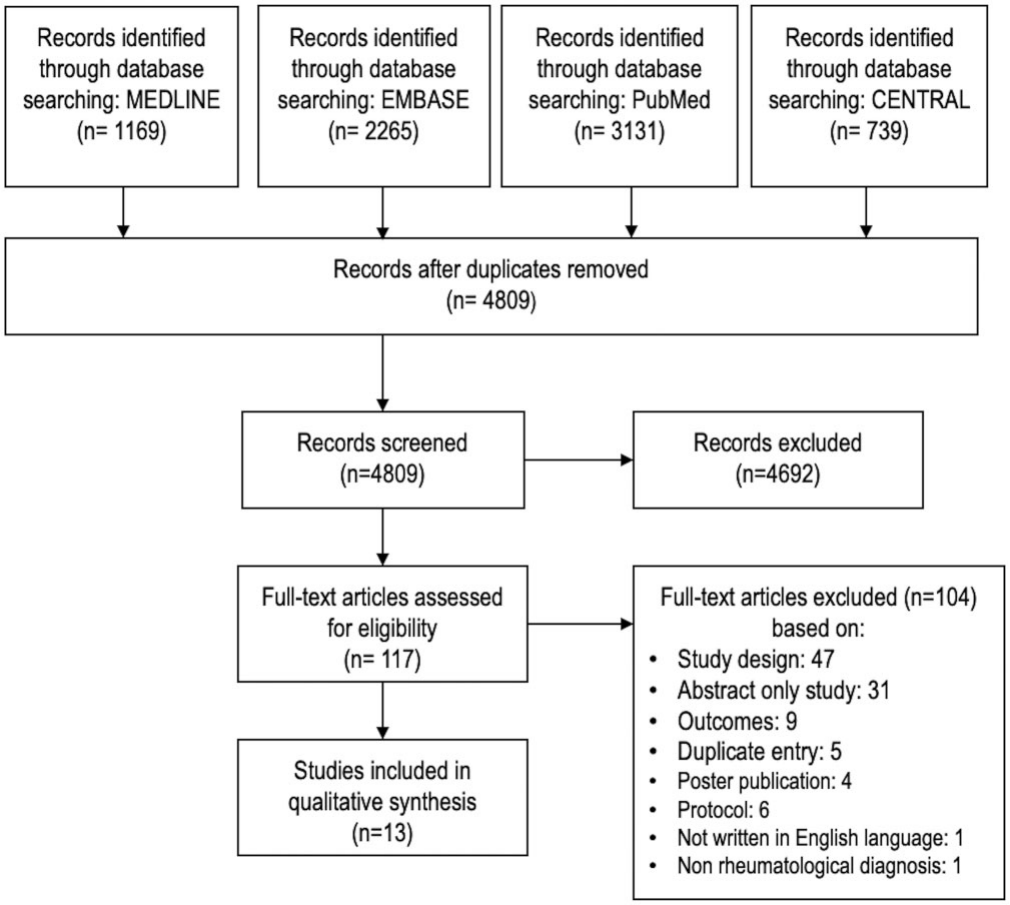
Preferred Reporting Items for Systematic Reviews and Meta-Analyses (PRISMA) flowchart of studies selected in the systematic review

The electronic databases identified 4809 non-duplicated abstracts for screening. Of these, 4692 were excluded based on title and abstract review. After retrieval of full-text publications, a further 104 publications were excluded, resulting in 13 papers included in this review (Fig. 1).

### Study characteristics

The characteristics of the 13 studies included in the review are summarized in Table 1. There were nine randomized controlled trials (RCTs), including one cross-over RCT, two prospective studies, one clinical controlled trial and one cross-sectional study. A total of 1451 patients were included across the 13 studies, and participant numbers ranged from 32 to 338 patients within studies. The studies were published between 2004 and 2020, with the majority (77%) of studies published from 2015 onwards (*n* = 10). Five studies (38%) included patients with RA alone, four studies included patients with mixed disease cohorts (31%), two studies (15%) included patients with OA, one study (8%) included patients with JIA, and one study (8%) included patients with FM.

**Table 1:** Baseline study characteristics

Author(s) (year)	Study design	Study period	Journal	Participant diagnoses	Location	Number of participants
Blixen *et al.* (2004) [20]	Randomized prospective study	March–June 1999	Journal of Telemedicine and Telecare	OA	USA	32
Pariser & O’Hanlon (2005) [12]	CCT	NS	Journal of Geriatric Physical Therapy	Mixed cohort (RA and OA)	USA	85
Stinson *et al.* (2010) [16]	RCT	October–November 2008	The Journal of Rheumatology	JIA	Canada	46
Poulsen *et al.* (2015) [22]	Prospective exploratory study	January–November 2012	International Journal of Rheumatic Diseases	Mixed cohort	Australia	108
Vallejo *et al.* (2015) [18]	RCT	NS	Journal of Psychiatric Research	FM	Spain	60
Kessler *et al.* (2016) [19]	Cross-sectional study	2014–2015	Paediatric Rheumatology	Mixed cohort	USA	338
Salaffi *et al.* (2016) [17]	RCT	NS	BioMed Central Musculoskeletal Disorders	Early RA	Italy	44
Ramelet *et al.* (2016) [13]	Crossover RCT	January 2010–August 2012	BioMed Central Paediatrics	Mixed cohort	Switzerland	55
Taylor-Gjevre *et al.* (2018) [21]	RCT	NS	Musculoskeletal Care	RA	Canada	85
O’Brien *et al.* (2018) [25]	RCT	May–June 2015	OA Research Society	Knee OA	Australia	120
de Thurah *et al.* (2018) [26]	RCT	May 2014–July 2015	Arthritis Care and Research	RA	Denmark	294
Zhao & Chen (2019) [15]	RCT	NS	Journal of Clinical Nursing	RA	China	92
Song *et al.* (2020) [14]	RCT	January–December 2015	Journal of Advanced Nursing	RA	China	92

CCT: controlled clinical trial; NS: not specified; RCT: randomized controlled trial.

### Patient characteristics

We contacted three authors for clarification regarding patient characteristics, eligibility criteria, telehealthcare models and outcome measures. Poulsen *et al.* [22] offered more information regarding the recruitment procedure for the study, method of telehealthcare, consultation method and grade of telehealthcare facilitator, and this was subsequently included in our review. No other authors were able to offer any further information for data analysis. Where data were available, the average age of patients included in the studies ranged from 13.1 to 64.4 years (Table 2). The majority of patients included in the studies were female sex, representing 38–100% of patients in 10 studies identifying patient sex. Recruitment procedures for studies varied throughout; however, the majority of patients were recruited from individual rheumatology departments. Two studies (15%) were conducted on patients who had been discharged after hospital admission for RA. The remaining studies were conducted on outpatients (Table 2). Table 3 describes the specific telehealthcare features of included studies. Table 4 summarizes the telehealthcare methods used throughout and the disease characteristics of patients included in the studies.

**Table 2:** Patient characteristics and recruitment procedures of included studies

Author(s) (year)	Age, mean (s.d.), years			Female sex, *n* (%)	Inclusion criteria	Recruitment procedures	Number of participants	Lost to follow-up, *n* (%)
	Overall	Control	Intervention					
Blixen *et al.* (2004) [20]	NS	69.9 (5.9)	71.7 (6.3)	12 (38)	Dx OA, ≥60 years old	Letter sent to patients seen in two rheumatology clinics in previous 6 months	32	2 (6)
Pariser & O’Hanlon (2005) [12]	64.4 (7.5)	NS	NS	68 (80)	Dx of RA or OA, 55years old	Patients from two rheumatology clinics	85	NS
Stinson *et al.* (2010) [16]	14.6 (1.5)	14.4 (1.3)	14.8 (1.7)	31 (67)	Dx JIA, English- and French-speaking adolescents aged 12–18 years who completed baseline online assessment	Patients from four tertiary care rheumatology clinics	46	6 (13)
Poulsen *et al.* (2015) [22]	54.2 (17–81)^a^	NS	NS	NS	NS	Patients invited at time of clinic appointment and offered a survey	108	0 0
Vallejo *et al.* (2015) [18]	51.6 (9.9)	53.5 (8.6)	49.8 (11.0)	60 (100)	Dx FM with adequate reading comprehension and access to computer, 18years old	Patients from one rheumatology unit in major city	60	7 (12)
Kessler *et al.* (2016) [19]	NS	NS	NS	NS	NS	Surveys offered to parents and guardians of children seen at a routine follow-up appointment in a paediatric rheumatology clinic	338	NS
Salaffi *et al.* (2016) [17]	NS	50.2 (16.3)	49.2 (15.2)	31 (70)	Dx RA, disease onset <1 year, CDAI ≥22, ≥18 years old	NS	44	3 (7)
Ramelet *et al.* (2016) [13]	13.1	NS	NS	NS	NS	Patients invited in rheumatology clinic of a tertiary referral hospital	55	3 (5)
Taylor-Gjevre *et al.* (2018) [21]	56.4 (11.5)	53.1 (12.2)	58.4 (10.7)	68 (80)	Dx RA who reside ≥100 km outside Saskatoon or Regina	Participants identified through Saskatoon rheumatology databases and invited via telephone call or clinic visit	85	31 (36)
O’Brien *et al.* (2018) [25]	NS	60.2 (13.9)	63.0 (11.1)	74 (62)	Dx knee OA, with knee pain lasting >3 months and average pain intensity score >3/10, classified as overweight or obese (BMI >27 or >40 kg/m^2^, respectively) and ≥18 years old	Patients on waiting list for outpatient orthopaedic consultation at a tertiary referral hospital	120	15 (13)
de Thurah *et al.* (2018) [26]	NS	60.7 (11.1)	60.5 (13.5)^b^ 61.6 (13.5)^c^	189 (64)	Dx RA >2 years, ≥18 years old and Danish language	All consecutive patients with a Dx of RA between May 2014 and July 2015 from two rheumatology clinics were invited to participate	294	19 (6)
Zhao & Chen (2019) [15]	55.5 (10.6)	54.2 (10.1)	56.9 (11.1)	66 (72)	Dx RA, ≥18 years old and discharged home from hospital	Patients from a university- affiliated and government hospital	92	15 (16)
Song *et al.* (2020) [14]	55.2 (10.8)	53.2 (10.0)	57.1 (11.3)	55 (60)	Dx RA ≥18 years old, discharged from hospital to home and able to speak Chinese	Patients discharged from department of rheumatology in tertiary care hospital	92	15 (16)

a Range. ^b^Age of patients receiving telemedicine intervention by rheumatologist. ^c^Age of patients receiving telemedicine intervention by nurse. CDAI: clinical disease activity index; Dx: diagnosis; NS: not specified.

**Table 3:** Telemedicine characteristics

Author(s), (year)	Telemedicine model	Method	Telehealthcare facilitator	Comparison	Reported outcomes	Duration of follow-up	Author conclusions	Effectiveness of intervention
Blixen *et al.* (2004) [20]	Telephone and audio delivered self-management	Six weekly health education models, mailings and relaxation audio tapes	ANP	Standard care	Primary: feasibility of self-management programme Secondary: QoL, SF36 survey, ASE scale, AIMS2 subscale, CES-D scale, satisfaction question	6 months	No significant difference between control and intervention	Non-inferior
Pariser & O’Hanlon (2005) [12]	Telephone- delivered self-efficacy advice	Five telephone calls over 6 weeks	NS	Standard care	Primary: ASE questionnaire Secondary: depression using geriatric depression scale, pain and fatigue scored 0–10 rating scale	6 weeks	Telephone intervention may assist older patients in managing arthritis	Non-inferior
Stinson *et al.* (2010) [16]	Website and telephone- delivered self-management	Restricted website-based management and telephone communication	Healthcare psychologist	Weekly telephone calls to discuss own efforts of self-management but no advice offered	Primary: HRQOL scale Secondary: recalled pain inventory, JIA specific knowledge MEPS questionnaire, severity of stress questionnaire, ASE scale, child adherence report questionnaire	12 weeks	Findings support feasibility and efficacy of Internet-based management programme for patients with JIA	Effective
Poulsen *et al.* (2015) [22]	Videoconferencing to monitor disease management	Questionnaire given after consultation	General and respiratory physician with rheumatology training	Face-to-face consultation	Questionnaire on quality of care and satisfaction	11 months	Patients satisfied with telemedicine service	Non-inferior
Vallejo *et al.* (2015) [18]	Website delivered CBT (iCBT)	Weekly access to materials, audio files and exercises	CBT group: clinical psychologist iCBT group: junior therapist under supervision of senior psychologist	CBT delivered face to face	Primary: FIQ score Secondary: HAD score, pain catastrophizing scale, chronic pain self-efficacy scale, chronic pain coping inventory	10 weeks	Internet-delivered iCBT is an appropriate method of reducing the impact of FM	Effective
Kessler *et al.* (2016) [19]	Videoconferencing follow-up	Questionnaires were delivered to parents and guardians of intervention and control groups after consultations	NS	Face-to-face consultation	Primary: questionnaire on distance travelled, amount of work and school missed, expenses	NS	Telemedicine clinics reduced the financial burden for patients previously travelling greater distances	Effective
Salaffi *et al.* (2016) [17]	Website- and telephone-delivered management	Telemonitoring of treatment strategy	NS	Face-to-face consultation at baseline, 3, 6, 9 and 12 months	Primary: disease activity assessed by CDAI at baseline and 1 year Secondary: comprehensive disease control measured using CDAI and ROAD at baseline and 1 year, erosive changes in hands and feet (radiographs) at baseline and 1 year	12 months	Telemedicine strategy leads to more effective disease remission and control	Effective
Ramelet *et al.* (2016) [13]	Videoconferencing follow-up	Videoconferencing sessions at rural sites with rheumatologist. Onsite physiotherapist present for examination reporting	Rheumatologist	Face-to-face consultation	Primary: disease activity DAS28-CRP score Secondary: QoL, satisfaction (VSQ9), patient global function score, RADAI, mHAQ	9 months	No significant difference between groups	Non-inferior
Taylor-Gjevre *et al.* (2018) [21]	Telephone-delivered consultation	Monthly telephone calls. Crossover trial: each group receives telenursing then standard care or vice versa	Two specialized nurses	Standard care	Primary: patient satisfaction (CSQ-8) Secondary: clinical health status (JAMAR)	24 months: 12 months in each strategy	Telenursing had a positive impact on satisfaction, morning stiffness and pain	Effective
O’Brien *et al.* (2018) [25]	Telephone consultation	Ten individually tailored telephone calls	Health-care professionals	Standard care	Primary: knee pain intensity reported using NRS Secondary: weight (in kilograms), knee disability (WOMAC scale), QoL (SF12v2), sleep quality (Pittsburgh sleep quality index), alcohol consumption, smoking prevalence, pain attitudes (SOPA), health-care utilization and emotional distress (DASS21)	26 weeks	Telephone consultations made no significant difference in reducing knee intensity compared with standard care	Non-inferior
de Thurah *et al.* (2018) [26]	Telephone consultation	3- to 4-monthly telephone calls	Four rheumatologists and four rheumatology nurses	Face-to-face consultation	Primary: disease activity assessed by DAS28 score Secondary: self efficacy (GSE), erosive changes (radiographs), HAQ, QoL (EQ5D)	52 weeks	Telephone consultation made no significant difference to disease activity	Non-inferior
Zhao & Chen (2019) [15]	Telephone consultation on health education	2^nd^, 4^th^, 8^th^ and 12^th^ week after hospital discharge	Two rheumatologist specialist nurses	One telephone consultation post-discharge: no advice offered, then standard care	Primary: self efficacy assessed by RASE score Secondary: disease activity (DAS28) and HAQ	24 weeks	Telephone consultations were beneficial in providing a health education programme to patients with RA	Effective
Song *et al.* (2020) [14]	Telephone consultation based on health-care education	12 week tailored intervention lasting 20–40 min, including four educational sessions	Nurses	Standard care from nursing staff post-discharge	Primary: disease activity assessed by ESR, CRP and DAS28 Secondary: medication adherence assessed by the Chinese version of the compliance questionnaire in rheumatology	24 weeks	Telephone education delivery improved medication adherence but had no impact on disease activity	Effective

ANP: advanced nurse practitioner; ASE: arthritis self-efficacy; CBT: cognitive behavioural therapy; CDAI: clinical disease activity index; CES-D: centre for epidemiological studies depression scale; DAS28: disease activity score 28; DASS21: depression and anxiety stress scale; FIQ: fibromyalgia impact questionnaire; GSE: generalized self-efficacy scale; HAD: hospital anxiety and depression scale; HRQOL: health-related quality of life; JAMAR: juvenile arthritis multidimensional assessment report; MEPS: medical exercise pain and social support; QoL: quality of life; mHAQ: modified HAQ; NS: not specified; RASE: RA self-efficacy; ROAD: recent onset disease activity index; SFS3: short form health survey; VSQ9 visit specific satisfaction; SF12v2: short form health survey; SOPA: survey of pain attitudes.

**Table 4:** Telehealthcare methods and participant diagnoses of included studies

	Number of studies	Number of patients	Percentage of total patients
Telehealthcare method, *n* = 588^a^			
Telephone	5	382	65
Website delivered	1	20	3
Videoconferencing	3	127	22
Mixed methods	3	59	10
Synchronous method facilitator, *n* = 588^a^			
Rheumatologist	1	54	9
Nurse	4	158	27
Rheumatologist and nurse	1	181	31
Other healthcare professional	4	150	26
Missing data	2	45	8
Diagnosis, *n* = 1451			
RA	5	607	42
OA	2	152	10
JIA	1	46	3
FM	1	60	4
Mixed cohort	4	586	40

a Study by Pariser *et al.* [12] was not included because numbers of participants in intervention and control group and facilitator grade were not available.

### Telehealthcare methods

Six studies (46%) used telephone consultation, three studies (23%) used mixed method communication, three studies (23%) used videoconferencing, and one study (8%) used website-delivered telecommunication as their method of telehealthcare delivery. The study by Pariser *et al.* [12] included a total number of 85 patients, but the numbers of patients receiving the telehealthcare intervention and the control intervention in the trial were not specified. The grade of the telehealthcare facilitator was also not identified in this study, which was therefore excluded from the analysis in Table 4.

### Telehealthcare effectiveness

Overall, the telehealthcare interventions in seven studies (54%) were deemed to be effective based on our interpretation of the conclusions of individual authors regarding the telehealthcare intervention. Effective telehealthcare interventions included three studies delivering self-management/self-efficacy advice through telephone consultations [13–15], two studies delivering self-management advice through website and telephone application [16, 17], one study delivering website-based cognitive behavioural therapy [18], and one study using videoconferencing follow-up clinics [19]. We identified non-inferiority of telehealthcare interventions in six studies (46%) when compared with standard care.

### Purpose of telehealthcare intervention and outcome measures of effectiveness 

Most of the studies used the telehealthcare intervention to provide a virtual consultation (*n* = 6, 46%). Three studies used the telehealthcare intervention to deliver a self-management programme (23%), two studies delivered a health education programme (15%), one study delivered a cognitive behaviour therapy intervention (8%), and one study delivered a self-efficacy programme (8%). In terms of outcome measures, there was vast variation in the primary outcome measures. Four studies measured the change in DAS using the ESR, CRP levels, disease activity score 28 (DAS28) and the clinical disease activity index. Two studies measured self-efficacy, defined in the study by Song *et al.* [14] as the ‘the degree of confidence in [the patient] performing a task’, and the remaining studies measured feasibility of the programme, quality of life, quality of care, impact of disease, convenience and expenses, pain intensity and satisfaction (Table 4).

### Telehealthcare facilitators

Two studies did not identify the telehealthcare facilitator grade, representing 8% of the telehealthcare intervention patients. Within available data, 31% of patients receiving telehealthcare intervention were managed by a both a rheumatologist and a nurse; however, this was represented from only one study. Four studies, representing 27% of patients receiving telehealthcare interventions, were managed by nurses, four studies used other health-care professionals as facilitators representing 26% of patients, and one study had a a rheumatologist alone as facilitator, representing 9% of patients.

### Patient satisfaction, cost effectiveness and attrition rates

Four studies reported patient satisfaction measures using patient satisfaction questionnaires. Overall, the four studies reported high satisfaction rates for patients receiving telehealthcare intervention. Blixen *et al.* [20] reported there was high patient satisfaction in both the telehealthcare intervention group and the standard care group; however, no significant difference was noted. The same outcome was reported by Taylor-Gjevre *et al.* [21]. Ramelet *et al.* [13] reported a progressive increase in patient satisfaction for those receiving telehealthcare intervention and a progressive decrease in patient satisfaction in those receiving standard care. Almost 90% of patients receiving the telehealthcare intervention in the study by Poulsen *et al.* [22] reported that they were highly satisfied with the service. Kessler *et al.* [19] included a cost-effectiveness measure and reported that standard care was associated with increased ancillary costs compared with the telemedicine clinic (92% *vs* 32%, *P* < 0.01). The attrition rate in studies ranged from 5 to 36%.

### Risk of bias

The quality of each study was assessed for risk of bias; 12 studies were assessed using the Cochrane Collaboration RoB 2, and one study was assessed using the NOS for cross-sectional studies. The risk-of-bias assessment for RCTs, prospective controlled studies and controlled clinical trials is summarized in Supplementary Fig. S1, available at *Rheumatology Advances in Practice* online, and the NOS assessment is detailed in Supplementary Table S2, available at *Rheumatology Advances in Practice* online. Overall, the risk of bias in included studies was predominantly high, with seven studies identified as high risk (Supplementary Fig. S2, available at *Rheumatology Advances in Practice* online). Four studies were identified as low risk of bias, and one study had ‘some concerns’ over the bias presented. High risk of bias was most commonly a result of the measurement of outcomes in studies and the randomization process of studies. The NOS identified the study by Kessler *et al.* [19] as moderate risk of bias with six stars.

## Discussion

This systematic review assessed the evidence of telehealthcare practices in rheumatology prior to the COVID-19 pandemic, to provide a baseline of evidence for the rapid change in rheumatological practice via telehealthcare application during the COVID-19 crisis. This is the first systematic review to address all forms of telehealthcare practices through all major disease cohorts of rheumatology (excluding trauma-related arthritis, drug reactions and infective rheumatic diseases).

There are two published accounts of systematic reviews on telehealthcare practices in rheumatology. Piga *et al.* [4] assessed the feasibility, effectiveness and patient satisfaction of telehealthcare practices and found weak evidence, based on the methodological bias of included studies, that ‘telerheumatology’ increased the feasibility and patient satisfaction rates. McDougall *et al.* [23] explored the use of ‘telerheumatology’ in the diagnosis and management of patients with inflammatory and autoimmune rheumatic diseases. More than half of the studies included in the review by McDougall *et al.* [23] were abstract-only reports or brief reports, precluding the validity of conclusions made and limiting the analysis of the review. The recent advances in telehealthcare practices throughout all health-care disciplines, particularly over the last 5 years, have contributed to further full-text publications regarding telehealthcare effectiveness in telehealthcare available for the present systematic review.

This systematic review demonstrates an array of telehealthcare methods studied across five countries worldwide. The most commonly studied telehealthcare method was telephone consultation, observed in six (46%) of the included studies. There were a range of disease cohorts studied, most commonly RA and mixed disease cohorts, reflecting real-life rheumatology clinical practice. Importantly, all of the studies included in this systematic review reported that telehealthcare interventions were effective or non-inferior to standard care in the respective measured outcomes of individual studies, which differed throughout. The present study included a range of disease cohorts, patient ages and telemedicine modes, which is reflective of the types of patients who would have been affected by the changes imposed by COVID-19, and therefore provides the most accurate representation for comparison of telehealthcare methods implemented during the COVID-19 pandemic. No study reported a detriment in health care in using telehealthcare models, validating the progressive adaptation to telehealthcare consultations more permanently post-COVID-19. Patient factors, including disease activity, cost effectiveness and patient satisfaction of the transition of care during COVID-19 crisis, must be assessed before any major and permanent changes to delivery of health care are made. It is important continually to reassess the outcomes of telehealthcare services, particularly during and after COVID-19.

COVID-19 has introduced a huge financial demand and burden to health-care systems worldwide [24]. There is not sufficient evidence of cost effectiveness reported in the included studies of the present review to infer that all telehealthcare methods are cost effective in patient-reported outcomes when compared with standard care. Additional studies regarding the cost effectiveness of telehealthcare and standard care are required, and ideally, the outcome measures of these future studies would be homogeneous to facilitate appropriate analysis. Measures of cost effectiveness are essential given the current economic crisis of COVID-19, particularly in rheumatology, where patient care has already been largely moved to telehealthcare methods by necessity [5].

The wide variety of measured outcomes of the included studies introduced methodological heterogeneity between the studies, limiting the conclusions drawn from this systematic review. Clinical diversity was evident, with variability in patient demographics, telehealthcare interventions and longevity of interventions, limiting the comparability of studies and summarizing of results. There was a considerable amount of missing data identified during data extraction, with only four studies providing all the proposed information [14, 16, 20, 25]. Missing data were evident throughout all the aforementioned areas of data extraction and must be considered when interpreting the results of the measured outcomes in the present systematic review. There may be considerable under/over-representation of percentage values were missing data have limited the analysis of outcome measures.

The majority of studies in this systematic review were identified as high risk of bias. There is a prerequisite bias evident in all studies of this systematic review, because it is impossible to enforce blinding given the nature of the interventions. Both the patient and the facilitator are aware of the interventions they have received, and there is no way of blinding the process. This introduces a potential bias and might affect both the quality of studies and the conclusions drawn.

The prevalence of telehealthcare has increased dramatically in recent years and has been applied in a variety of health-care disciplines; however, the uptake of telehealthcare application in rheumatology has been fairly minimal [4]. Examinations are key to diagnosis and management of rheumatological disease, presenting a major limitation to telehealthcare practice. There are limited data on the validity of remote examination during telehealthcare consultation when compared with standard care [27]. One of the studies in the present review included an onsite examiner for patients receiving the telehealthcare intervention [21]. Although the telehealthcare intervention in this study was deemed non-inferior to face-to-face consultation, we must consider the effect that on-site examination might have had on the measured outcomes of this study. Hybrid models of telehealthcare include an on-site examination carried out by a trained professional and a telemedicine consultation in synchrony. Hybrid telehealthcare models might be a solution to the limitations of examination during telehealthcare consultations and could improve efficiency of the consultation and provide patient-centred care. There is an inevitable lack of equity and uniformity within telehealthcare consultations, which might exclude patients without technological hardware, without technical skills and education, with hearing and sight impairments, with language barriers and with disabilities. The application of telehealthcare services to rheumatology must be tailored to individual patient needs and considerate of factors that might present difficulties to the implementation of telehealthcare delivery.

### Conclusion

COVID-19 has rapidly changed the direction of management in rheumatology, leading to a vastly increased number of telehealthcare consultations during the global pandemic. Most of the studies in the present review were used to provide a virtual consultation, most commonly by telephone; however, other interventions included health education, self-management and self-efficacy through a range of media and modes. All telehealthcare interventions were deemed either effective or non-inferior to standard care with regard to the outcome measures of individual studies. The extent of the effectiveness of telehealthcare methods prior to COVID-19 is unclear, because the evidence for the use of telehealthcare in rheumatology is lacking and there are wide variations in the outcome measures studied. The evidence of effectiveness is limited by the methodological bias and clinical heterogeneity of telehealthcare interventions, preventing definitive inferences. With a paradigm shift in the nature of patient consultation, assessment fit for purpose is essential. Scrutinous assessment of the current telehealthcare interventions used during COVID-19 is required to accommodate recommendations and guideline reviews directed from international working groups. Future research is needed to clarify the effectiveness of telehealthcare interventions, particularly after COVID-19 and should focus on cost effectiveness, disease management and patient satisfaction. Defining the uptake and response of patients within different age groups receiving telehealthcare interventions in rheumatology during the COIVD-19 pandemic would also be an interesting research point for future studies.

*Funding:* No specific funding was received by any bodies in the public, commercial or not-for-profit sectors to carry out the work described in this article.

*Disclosure statement:* The authors have declared no conflicts of interest.

## Data availability statement

The data underlying this systematic review are available within the article and in its online supplementary material.

## Supplementary data

Supplementary data are available at *Rheumatology Advances in Practice* online.

## Supplementary Material

rkab073_Supplementary_Data
