# Supplementary material for: A systematic review exploring pre-COVID-19 telehealthcare models used in the management of patients with rheumatological disease
Source: Rheumatol Adv Pract. 2021 Nov 13;5(3):rkab073. doi: 10.1093/rap/rkab073 (PMC8599884; doi:10.1093/rap/rkab073)
Supplement: rkab073_Supplementary_Data [file rkab073_supplementary_data.zip › 20-140 Supplementary Material.docx]

**Supplementary Table S1:** Search strategy used to identify papers in this review using the Healthcare Database Advanced Search engine.

| **Telehealthcare terms** | **Rheumatology terms** |
| --- | --- |
| exp TELEMEDICINE/  telemedicine  telehealth  telecommunication  telehealthcare  tele consult  tele* consult*  phone* consult*  telemed*  remote consult*  remote communicat*  remote access  remote management  telecare  videoteleconfer*  VTC  Ehealth  Econsult  Interactive  Video confer*  Twoway  Asynchronous consult  Synchronous consult | rheumatic  rheumatoid  rheumatology  rheum* clinic  arthritis  polyarthritis  crystal arthropath* gout  SLE  Systemic erythematous lupus  Diffuse systemic sclerosis  Limited systemicsclerosis  SSc  Scleroderma  Rheumatic disease  Polymyositis  Dermatopolymtositis  Joint disease  Rheumatoid arthritis  RA  Osteoarthritis  OA  Non?articular rheum*  Miscellaneous disorder  Connective tissue disease  CTD connective tissue  Collagen disorder  Collagen disease  Arteritis  Polyarteritis nodosa  PAN  Giant cell arteritis  GCA erythema nodosum  JIA  Juvenile idiopathic arthritis  Reiters syndrome |

| **Supplementary Table S2**: Newcastle Ottawa Scale for assessment of bias for cross sectional studies | | | | | | | | |
| --- | --- | --- | --- | --- | --- | --- | --- | --- |
| Author, year | **Selection (max 5*)** | | | | **Comparability (max 2*)** | **Outcome (max 3*)** | | **Overall** |
|  | Representativeness of sample | Sample size | Non-respondents | Ascertainment of exposure |  | Assessment of the outcome | Statistical test |  |
| Kessler et al., 2015 |  |  | * | ** | * | * | * | 6* |
| *represents the number of stars appointed after assessment | | | | | | | | |

**Supplementary Figure S1:** A summary of the risk of bias across the randomised studies, assessed using the revised Cochrane Risk of Bias 2 assessment tool.


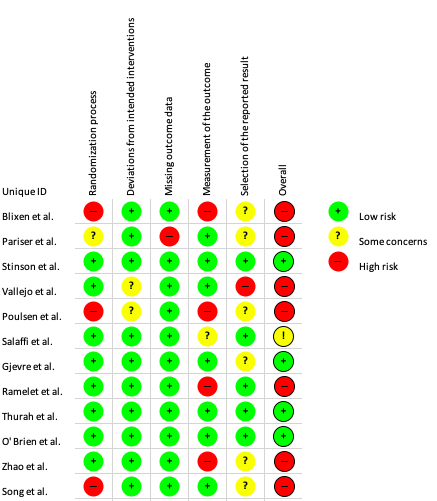


**Supplementary Figure S2:** A summary of risk of bias within individual randomised studies, assessed using the revised Cochrane Risk of Bias 2 assessment tool.
